# Effect of remimazolam *vs.* propofol on hemodynamics during general anesthesia induction in elderly patients: Single-center, randomized controlled trial

**DOI:** 10.7555/JBR.37.20230110

**Published:** 2023-11-15

**Authors:** Mingfeng He, Chanjuan Gong, Yinan Chen, Rongting Chen, Yanning Qian

**Affiliations:** Department of Anesthesiology and Perioperative Medicine, the First Affiliated Hospital of Nanjing Medical University, Nanjing, Jiangsu 210029, China

**Keywords:** remimazolam, propofol, elderly patients, hypotension, left ventricular systolic function, systematic vascular resistance

## Abstract

The current study aimed to compare the effects between remimazolam and propofol on hemodynamic stability during the induction of general anesthesia in elderly patients. We used propofol at a rate of 60 mg/(kg·h) in the propofol group (group P) or remimazolam at a rate of 6 mg/(kg·h) in the remimazolam group (group R) for the induction. A processed electroencephalogram was used to determine whether the induction was successful and when to stop the infusion of the study drug. We measured when patients entered the operating room (T_0_), when the induction was successful (T_1_), and when before (T_2_) and 5 min after successful endotracheal intubation (T_3_). We found that mean arterial pressure (MAP) was lower at T_1–3_, compared with T_0_ in both groups, but higher at T_2_ in the group R, while ΔMAP_T0–T2_ and ΔMAP_max_ were smaller in the group R (ΔMAP_T0–T2_: the difference between MAP at time point T_0_ and T_2_, ΔMAP_max_: the difference between MAP at time point T_0_ and the lowest value from T_0_ to T_3_). Cardiac index and stroke volume index did not differ between groups, whereas systemic vascular resistance index was higher at T_1–3_ in the group R. These findings show that remimazolam, compared with propofol, better maintains hemodynamic stability during the induction, which may be attributed to its ability to better maintain systemic vascular resistance levels.

## Introduction

Hypotension during the induction of general anesthesia occurs frequently, which is usually defined as a decrease of ≥ 20% in systolic blood pressure (SBP) from baseline or ≤ 80 mmHg, or a decrease in mean arterial pressure (MAP) to ≤ 60 mmHg. Hypotension can lead to kidney or myocardial damage, extend the length of stay in the intensive care unit, require postoperative mechanical ventilation, and increase perioperative complications and mortality^[1]^. Elderly patients are especially vulnerable to these negative outcomes^[2]^.

Remimazolam is a novel, ultrashort-acting benzodiazepine with the advantages of a rapid onset, an organ-independent metabolism, and a lack of accumulation after long-term infusion^[3–4]^. Remimazolam has been widely used for procedural sedation in patients undergoing gastroenteroscopy, colonoscopy, or hysteroscopy^[4–5]^. Remimazolam has been approved for the induction of general anesthesia in Japan and South Korea since 2020^[6]^, and it has become a commonly used anesthesia induction drug in clinical work and research^[7]^. Although it is believed that the use of remimazolam and propofol for the induction of general anesthesia can lead to a decrease in blood pressure (BP), studies on the differences between the effects of remimazolam and propofol on BP are not entirely consistent^[8–9]^. Moreover, studies examining the effects on both left ventricular systolic function and systemic vascular resistance (SVR) during induction have yielded conflicting results^[10–11]^.

Considering that propofol and remimazolam are two commonly used agents in clinical practice, we sought to compare the effects of the two drugs on hemodynamics in elderly patients, using a titrated method of administration for the induction of general anesthesia.

## Materials and methods

### Ethics and trial registration

This was a single-center, prospective, randomized controlled trial. Ethical approval for the current study (No. 2022-SR-036) was obtained from the Ethics Committee of the First Affiliated Hospital of Nanjing Medical University on March 29, 2022. The trial was registered in the Chinese Clinical Trial Registry before patient enrollment (http://www.chictr.org.cn/showproj.aspx?proj=167328; No. ChiCTR2200059697; principal investigator: He Mingfeng; date of registration: May 8, 2022). A written informed consent was obtained from each participant.

### Inclusion and exclusion criteria

Elderly patients aged 65 years or older with an American Society of Anesthesiologists (ASA) physical status of Ⅱ or Ⅲ, and a body mass index (BMI) between 19 and 25 kg/m^2^, who underwent elective transurethral minimally invasive surgery under general anesthesia at the First Affiliated Hospital of Nanjing Medical University between May 16, 2022 and September 02, 2022 were recruited.

Patients were excluded if they met any of the following exclusion criteria: 1) an abnormal liver function, defined as aspartate aminotransferase or alanine aminotransferase > 2.5× the upper limit of normal, or a medical history of hepatectomy or liver transplantation^[8]^; 2) an abnormal renal function (serum creatinine level > 2 mg/dL)^[12]^; 3) a high probability of difficult airway or mask ventilation; 4) severe cardiovascular disease, including sick sinus syndrome, a medical history of myocardial infarction, or uncontrolled severe hypertension, defined as SBP > 180 mmHg or diastolic blood pressure (DBP) ≥ 110 mmHg; or 5) a history of prolonged use of sedatives, sleeping pills, antidepressants, or a heavy alcohol consumption before surgery.

### Randomization and masking

#### Randomization

The research group consisted of three investigators (*i.e.*, investigator-1, investigator-2, and investigator-3). Eligible patients were randomized into either the "propofol group" (group P) or the "remimazolam group" (group R) according to a random digit table generated with SPSS 26.0 (SPSS Inc., Chicago, IL, USA) by investigator-2.

#### Masking

Because propofol (milky white) and remimazolam (clear and colorless) differ in appearance, the study design was not completely double-blind. However, investigator-1 and investigator-3 were blinded to the patient allocation throughout the study. Investigator-2 was aware of the patient group but was not allowed to communicate relevant information to the patients, investigator-1, or investigator-3. In addition, investigator-2 was required to cover the intravenous infusion line to prevent investigator-1 and investigator-3 from making any assumptions.

### Anesthesia

All patients underwent routine fasting without preoperative medication. In addition to standard monitoring, processed electroencephalogram values were simultaneously monitored using the Bispectral Index™ (BIS™) Monitoring System (BIS, Medtronic, Minneapolis, MN, USA) after patients entered the operating room. Noninvasive BP was measured after 5 min of rest. Patients with an SBP ≥ 180 mmHg and/or a DBP ≥ 110 mmHg were excluded from the study. After successful radial artery catheterization, a blood gas analysis was performed, and the patients with a severe acid-base balance (pH values < 7.30 or > 7.50) or electrolyte disturbances (K^+^ ≤ 2.5 mmol/L or K^+^ ≥ 5.3 mmol/L; Na^+^ ≤ 130 mmol/L or Na^+^ ≥ 150 mmol/L) were also removed. Echocardiography was performed by investigator-3, a physician who had been practicing anesthesia in cardiovascular surgery for over 10 years and had received formal training and certification in transthoracic and transesophageal cardiac ultrasound. Investigator-1 was responsible for collecting data in addition to ultrasound results and providing appropriate medical advice. Investigator-2 administered the corresponding drug according to the study plan and the instructions of investigator-1.

After at least 15 min of rest (T_0_), BP, heart rate (HR), pulse oxygen saturation (SpO_2_), and BIS values were recorded, and a transthoracic echocardiography (TTE) was performed. Anesthesia induction was initiated after pre-oxygenation at an oxygen flow rate of 6 L/min for at least 5 min. According to the instruction manuals of propofol and remimazolam and the study of Chen L *et al*^[13]^, the drug was pumped intravenously at a rate of 6 mg/(kg·h) for remimazolam tosylate (Jiangsu Hengrui Pharmaceutical Co., Ltd., Lianyungang, Jiangsu, China) in the group R and 60 mg/(kg·h) for propofol (Aspen Pharma Trading., Ltd., Dublin, Ireland) in the group P, respectively^[8,12]^. When a patient lost consciousness (defined as the patient not responding to a tap on the shoulder)^[12]^, the mandible was gently lifted to open the airway without artificial assistance or mechanical ventilation.

When the BIS value decreased to 60 (T_1_), the TTE was performed. At the same time, *cis*-atracurium (0.15 mg/kg) and sufentanil (0.5 μg/kg) were administered, and propofol was pumped at a rate of 4 to 10 mg/(kg·h) and remimazolam was pumped at a dose of 0.5 to 2 mg/(kg·h). Mechanical ventilation was initiated in the absence of respiratory movements. The pressure-limiting ventilation mode was used before endotracheal intubation to prevent unstable airway pressure due to volume-controlled ventilation, which may affect the accuracy of ultrasound examinations^[14]^. The parameters were set as follows: peak airway pressure, 12 cm H_2_O; respiratory rate, 10 breaths/min; inspiratory respiration ratio, 1∶2; and inhalation oxygen concentration, 100%. TTE was performed 4 min after the intravenous administration of *cis*-atracurium (T_2_), followed by endotracheal intubation. Another TTE was performed 5 min after endotracheal intubation (T_3_).

If there was a significant "retraction sign of three fossae" during inspiration, or if SpO_2_ was < 90% before loss of consciousness (LoC), the patient's jaw was gently lifted, or an assisted breathing was performed, as needed. Ephedrine (6 mg) or phenylephrine (50 to 100 μg) was administered intravenously when the BP dropped by more than 30% of the baseline value or when the MAP was < 65 mmHg. Atropine (0.5 mg) was injected intravenously when the HR fell below 50 beats/min for > 1 min, and esmolol (20 mg) was administered intravenously when the HR fell below 100 beats/min.

**Figure 1:**
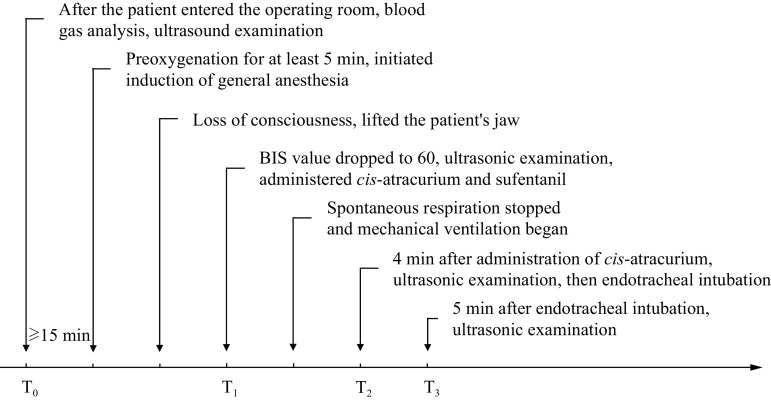
General anesthesia process, experimental intervention, and data collection time points.

The entire process, including general anesthesia, experimental intervention, and data collection, is illustrated in ***Fig. 1***.

### Determining cardiac index (CI), stroke volume index (SVI), and systematic vascular resistance index (SVRI) using TTE

**Figure 2:**
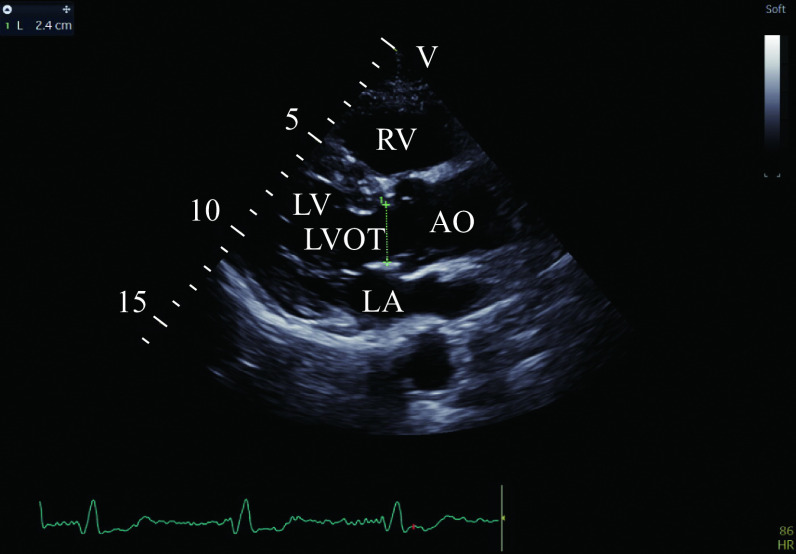
Measurement of LVOTd.

All echocardiographic measurements were performed by investigator-3 according to the American Society of Echocardiography/European Association of Cardiovascular Imaging guidelines. Comprehensive two-dimensional echocardiography, Doppler, and color Doppler examinations were performed using a GE Vivid E95 echo scanner (GE Healthcare, Milwaukee, WI, USA) equipped with an M5S electronic phased array probe (frequency 1.5–4.0 MHz). The left ventricular outflow tract diameter (LVOTd) was determined based on the parasternal long-axis view when the systolic aortic valve was fully opened (***Fig. 2***).

**Figure 3:**
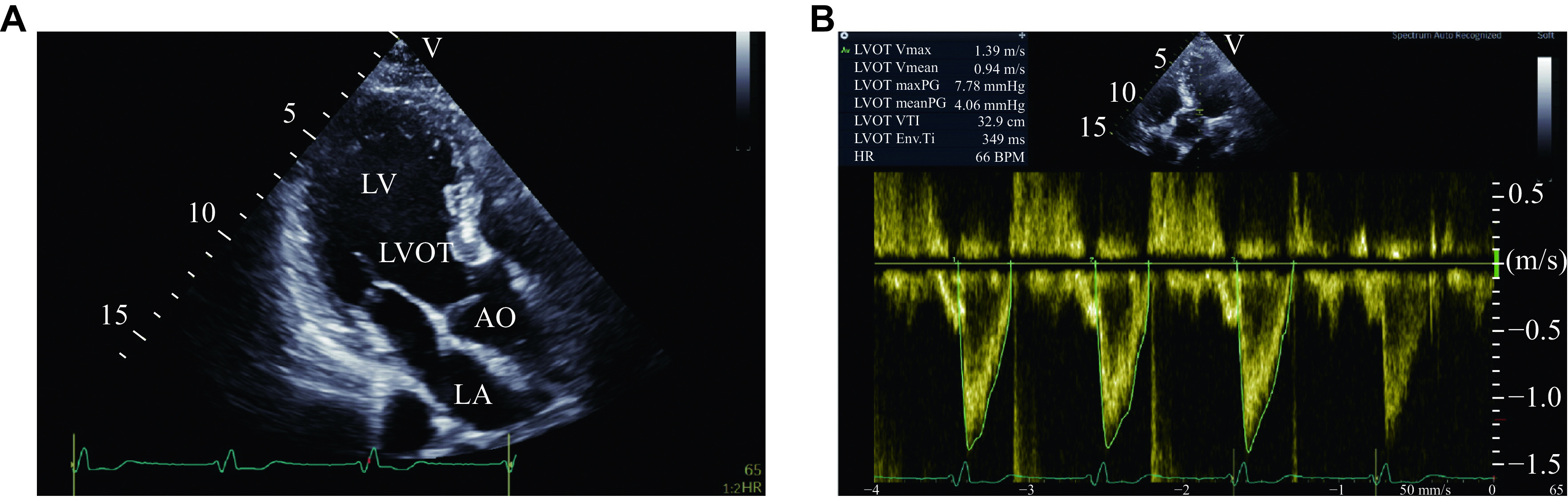
Measurement of VTI_LVOT._

By placing the sample volume in the left ventricular outflow tract in the apical three-chamber view (***Fig. 3A***), the left ventricular outflow tract velocity-time integral (VTI_LVOT_) was measured in pulsed Doppler mode (***Fig. 3B***).

The LVOTd and VTI_LVOT_ values were then determined for each patient using images saved during the induction by investigator-3, and other indicators were calculated according to the following formulas:

1 $ {{\mathrm{BSA}}}=0.0061 \times {{\mathrm{H}}}+0.0128 \times {{\mathrm{W}}}-0.1529 $

2 $ \mathrm{SV}=\mathrm{VTI}_{\mathrm{LVOT}} \times \pi \times\left(\frac{\mathrm{LVOTd}}{2}\right)^2, $

3 $ \mathrm{SVI}=\frac{\mathrm{SV}}{\mathrm{BSA}}, $

4 $ \mathrm{CI}=\frac{\mathrm{CO}}{\mathrm{BSA}}=\frac{\mathrm{SV} \times \mathrm{HR}}{\mathrm{BSA}}, $

5 $ \mathrm{SVR}=80 \times \frac{\mathrm{MAP}}{\mathrm{CO}}\; {\mathrm{and}} $

6 $ \mathrm{SVRI}=\mathrm{SVR} \times \mathrm{BSA}. $

BSA indicates the body surface area (m^2^), H indicates the height of patient (cm), W indicates the weight of patient (kg), SV indicates the stroke volume, SVI indicates the stroke volume index, CI indicates the cardiac index, CO indicates the cardiac output, SVR indicates the systematic vascular resistance, and SVRI indicates the systematic vascular resistance index.

### Outcomes

#### Primary outcomes

 The primary outcomes were ΔMAP, defined as the difference between MAP at time point T_0_ and time points T_1_, T_2_, or T_3_, and ΔMAP_max_, defined as the difference between MAP at time point T_0_ and the lowest value of MAP at any time from T_0_ to T_3_.

#### Secondary outcomes

The CI, SVI, and SVRI at each time point as well as the incidence of changes in BP and HR that needed to be addressed during induction were recorded. In addition, the following indicators were also recorded: the time and dose/weight required for LoC and for BIS values to drop to 60, and BIS value at LoC and eye-opening.

### Statistical analysis

#### Calculation of minimum sample size

The minimum sample size was calculated through a preliminary experiment performed after the ethics approval was obtained. The results of eight patients included in each group of this preliminary experiment yielded a mean difference in MAP between the base value and the moment when the BIS decreased to 60 of 5.7 and a pooled standard deviation of 5.7. To achieve a power of 0.8 and an alpha error of less than 0.05, we estimated that 47 patients were needed for the main trial. To allow for dropouts, 30 patients were randomly assigned to each group.

#### Statistic analysis of data

Data were analyzed using SPSS version 26.0. Normality and homogeneity of variance were analyzed using the Kolmogorov-Smirnov and Levene's tests. Data that conformed to a normal distribution were expressed as the mean ± standard deviation and analyzed using Student's *t*-test. Chi-square (*χ*^2^) test, continuity correction *χ*^2^ test, and Fisher's exact test were used to compare categorical data between the two groups. Rank data were described as numbers and compared using the Wilcoxon rank-sum test. Two-way analysis of variance (ANOVA) and Tamhane's T2 test were used to compare the measurement data of multiple groups according to whether data variances were equal. Two-sided *P* values of < 0.05 were considered statistically significant for all tests.

## Results

### Study participants

**Figure 4:**
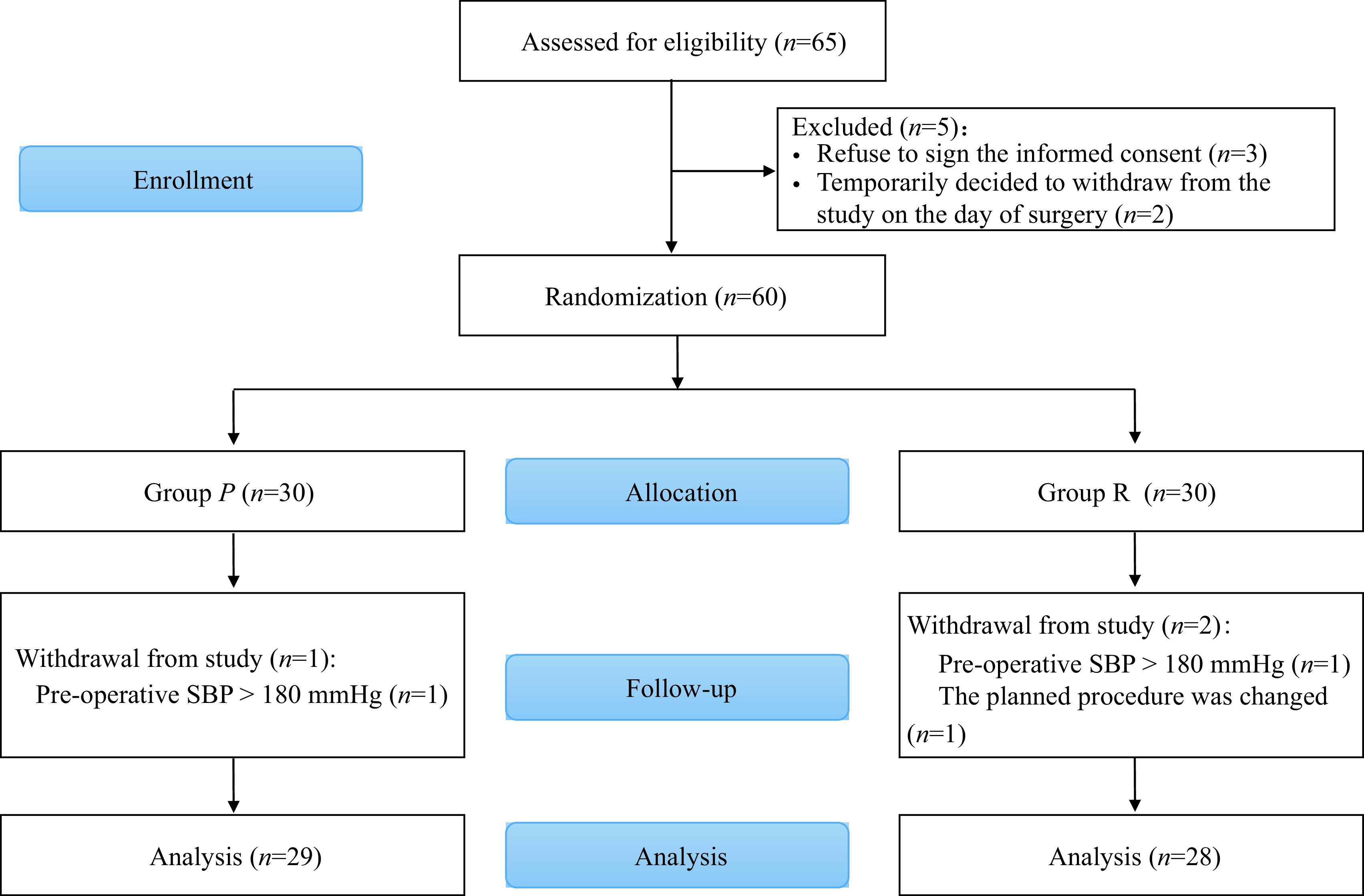
Enrollment, allocation, follow-up, and data analysis.

A total of 65 patients were initially recruited to participate in the study, but five patients were excluded (three patients refused to sign the informed consent form, and two patients withdrew on the day of surgery). Of the 60 recruited patients, three dropped out of the study (two due to a high preoperative SBP and one due to a change in surgical procedure). Twenty-nine patients in the group P and 28 patients in the group R were included in the final analysis (***Fig. 4***).

### Demographic and baseline characteristics

***Table 1*** shows demographic and baseline characteristics of all patients, with no significant differences between the group P and the group R (*P* > 0.05).

**Table 1:** Demographic and baseline characteristics

Characteristics	Group P^a^ (*n*=29)	Group R^a^ (*n*=28)	Statistics^b^	*P-*value
Age (years)	70.8±3.5	70.3±4.1	*t*=0.536	0.594
Height (cm)	168.0±8.1	167.4±5.4	*t*=0.331	0.742
Weight (kg)	65.1±8.4	64.3±5.3	*t*=0.418	0.677
BMI (kg/m^2^)	23.0±1.6	22.9±1.2	*t*=0.117	0.907
Sex (male/female)	24/5	26/2	*χ*^*2*^=0.574	0.449
ASA grade (Ⅱ/Ⅲ)	24/5	25/3	*Z*=−0.703	0.482
Type of surgery (1/2/3)^c^	16/8/5	14/7/7	*χ*^*2*^=0.516	0.773
SpO_2_ (%)	97.2±1.4	97.5±1.3	*t*=−0.799	0.428
Hb (g/dL)	13.3±1.2	13.0±1.4	*t*=0.967	0.338
Total protein (g/L)	67.5±6.8	69.7±6.5	*t*=−1.241	0.220
Albumin (g/L)	38.6±3.6	40.1±4.3	*t*=−1.456	0.151
Creatinine (μmol/L)	72.7±19.5	70.1±12.6	*t*=0.586	0.560
Urea nitrogen (mmol/L)	6.46±1.69	5.90±1.22	*t*=1.451	0.155^d^
ALT (U/L)	16.4±7.7	20.0±8.9	*t*=−1.622	0.111
AST (U/L)	20.1±4.1	23.7±8.6	*t*=−2.004	0.052^d^
Preoperative complication (yes/no)				
Hypertension	10/19	9/19	*χ*^*2*^=0.35	0.851
DM	6/23	3/25	*χ*^*2*^=0.448	0.503
Myocardial ischemia^e^	3/26	4/24	*χ*^*2*^=0.002	0.960
Conduction block	3/26	6/22	*χ*^*2*^=0.615	0.433
^a^Eligible patients were randomized into either the "propofol group" (group P) or the "remimazolam group" (group R).^b^Data that conformed to a normal distribution were expressed as the mean ± standard deviation and analyzed using Student's *t*-tests. Rank data (ASA grade) were described as numbers and compared using the Wilcoxon rank-sum test. The Chi-square (*χ*^*2*^) tests (type of surgery and hypertension) and continuity correction *χ*^*2*^ test (sex, DM, myocardial ischemia, and conduction block) were used to compare categorical data.^c^Type of surgery: 1 indicated transurethral laser resection of the prostate, 2 indicated special treatment for transurethral bladder tumors, and 3 indicated ureteroscopic lithotripsy, cystoscopic urethra, or other procedures, respectively.^d^The variances in plasma urea nitrogen and AST levels were not equal between the two groups (Levene's variance equality test, *P*_nitrogen_ = 0.024, *P*_AST_ = 0.003).^e^Patients at high risk for myocardial ischemia included those diagnosed with coronary heart disease after coronary angiography or dual-source CT who did not meet the criteria for coronary stenting or coronary artery bypass grafting.Abbreviations: BMI, body mass index; ASA, American Society of Anesthesiologists; SpO_2_, pulse oxygen saturation; Hb, plasma hemoglobin concentration; ALT, alanine aminotransferase; AST, aspartate aminotransferase; DM, diabetes mellitus.				

### Comparison of MAP, HR, and BIS values

**Figure 5:**
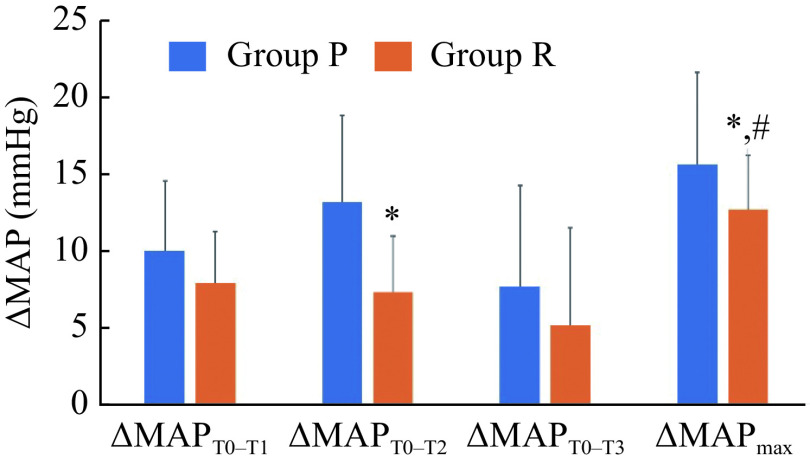
Comparison of ΔMAP.

MAP and BIS values were lower at time points T_1_, T_2_, and T_3_ in both groups than those at time point T_0_ (*P* < 0.05). Compared with the group P, MAP was higher at T_2_ in the group R (*P* < 0.05), whereas the differences were not statistically significant at T_0_, T_1_, and T_3_ (*P* > 0.05). There were no significant differences in HR across time points T_0_–T_3_ in either group (*P* > 0.05, ***Table 2***). ΔMAP_T0–T2_ and ΔMAP_max_ were lower in the group R (*P* < 0.05, ***Fig. 5***) than in the group P.

**Table 2:** Comparison of MAP, HR, and BIS values between groups

Time points	MAP (mmHg)				HR (BPM)				BIS		
	Group P^a^ (*n*=29)	Group R^a^ (*n*=28)	*P-*value		Group P^a^ (*n*=29)	Group R^a^ (*n*=28)	*P-*value		Group P^a^ (*n*=29)	Group R^a^ (*n*=28)	*P-*value
T_0_^b^	93.8±9.0	92.3±10.8	0.559		70.6±10.7	70.4±9.0	0.963		95.6±1.4	96.1±1.4	0.173
T_1_	83.8±8.4^b^	84.4±9.2^b^	0.81		67.0±8.3^b^	65.6±7.6^b^	0.522		60^c^	60^c^	–^c^
T_2_^b^	80.7±7.3	85.0±8.7	0.048^*^		63.1±5.8	62.5±5.7	0.661		49.0±3.6	51.0±4.3	0.062
T_3_^b^	86.2±5.9	87.1±7.5	0.618		69.0±8.2	67.8±7.1	0.542		47.3±6.9	49.7±4.4	0.125
^a^Eligible patients were randomized into either the "propofol group" (group P) or the "remimazolam group" (group R).^b^BIS values at time points T_0_, T_2_ and T_3_, as well as MAP and HR at each time point were normally distributed, expressed as mean ± standard deviation, and analyzed using Student's *t*-tests. ^*^P < 0.05, compared with the group P.^c^Because T_1_ was defined as the BIS value decreased to 60, it was a constant in the table and no statistical analysis was performed. Abbreviations: MAP, mean arterial pressure; HR, heart rate; BIS, bispectral index; T_0_, after the patient entered the operation room and rested for at least 15 min; T_1_, BIS value decreased to 60 after the initiation of induction; T_2_, 4 min after administration of *cis*-atracurium; T_3_, 5 min after endotracheal intubation.											

### Comparison of left ventricle systolic function

There were no significant differences in CI and SVI between the two groups at any time point (*P* > 0.05, ***Table 3***).

**Table 3:** Comparison of CI and SVI

Time points	CI [L/(min·m^2^)]				SVI [mL/(beat·m^2^)]		
	Group P^a^ (*n*=29)	Group R^a^ (*n*=28)	*P-*value		Group P^a^ (*n*=29)	Group R^a^ (*n*=28)	*P-*value
T_0_	2.93±0.41	2.99±0.46	0.617		42.4±8.5	42.8±6.2	0.852
T_1_	2.96±0.33	2.80±0.46	0.122		44.7±6.2	43.1±7.8	0.389
T_2_	3.09±0.32	3.04±0.47	0.639		49.2±5.9	48.8±7.1	0.776
T_3_	3.35±0.55	3.16±0.81	0.320^b^		48.8±7.8	47.0±13.0	0.544^b^
^a^Eligible patients were randomized into either the "propofol group" (group P) or the "remimazolam group" (group R). In both groups, CI and SVI at each time point were normally distributed, expressed as mean ± standard deviation, and analyzed using Student's *t*-tests.^b^Variances of ΔMAP_max_ were not equal between the two groups (Levene's variance equality test, *P*_CI_ at T_3_ = 0.008, *P*_SVI_ at T_3_ = 0.008).Abbreviations: CI, cardiac index; SVI, stroke volume index; T_0_, after the patient entered the operation room and rested for at least 15 min; T_1_, BIS value decreased to 60 after the initiation of induction; T_2_, 4 min after administration of *cis*-atracurium; T_3_, 5 min after endotracheal intubation.							

### Comparison of SVRI

**Figure 6:**
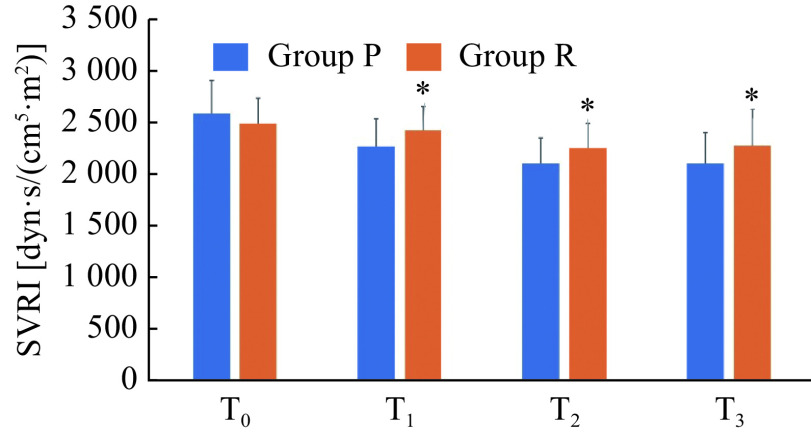
Comparison of SVRI.

At T_0_, there was no significant difference in SVRI between the two groups (*P* > 0.05). At time points T_1_, T_2_, and T_3_, the SVRI was higher in the group R than in the group P (*P* < 0.05, ***Fig. 6***).

### Comparison of factors that affect sedative dose

The time required for LoC, the time required for the BIS value to drop to 60, and the time from LoC to the BIS value reaching 60 were longer in the group R (*P* < 0.01, ***Table 4***). The dose/weight required for LoC was 1.51 (± 0.37) mg/kg in the group P but 0.18 (± 0.03) mg/kg in the group R, while the dose/weight required for the BIS value to drop to 60 was 1.88 (± 0.41) mg/kg in the group P but 0.26 (± 0.04) mg/kg in the group R, and these differences were all statistically significant (***Table 4***).

**Table 4:** Comparison of factors that affect sedative dose

Characteristics	Group P^a^ (*n*=29)	Group R^a^ (*n*=28)	*t*-test	*P-*value
Time required for LoC (s)	90.8±22.4	106.0±18.9	−2.753	0.008^**^
Time required for BIS=60 (s)	112.5±24.4	154.2±22.2	−6.728	< 0.001^**^
Time from LoC to BIS=60 (s)	21.7±7.1	48.2±12.9	−9.560	< 0.001^b,**^
Dose/weight required for LoC (mg/kg)	1.51±0.37	0.18±0.03	19.156	< 0.001^b,**^
Dose/weight required for BIS=60 (mg/kg)	1.88±0.41	0.26±0.04	21.341	< 0.001^b,**^
BIS value at LoC	67.5±4.0	67.9±5.0	−0.313	0.755
BIS value at eye-opening	75.9±7.6	72.7±6.7	1.685	0.098
^a^Eligible patients were randomized into either the "propofol group" (group P) or the "remimazolam group" (group R). Data in both groups were normally distributed, expressed as mean ± standard deviation, and analyzed using Student's *t*-tests.^b^Variances were not equal between the two groups (Levene's variance equality test, *P* < 0.05). ^**^P < 0.01, compared with the group P. Abbreviations: LoC, loss of consciousness; BIS, bispectral index.				

There were no significant differences in BIS values at LoC or eye-opening between the two groups (*P* > 0.05, ***Table 4***).

### Comparison of complication rates

Patients in the group R had a lower incidence of injection pain (*P* < 0.001). However, there were no statistically significant differences between the two groups in terms of hypotension, bradycardia, hypertension, or hypoxemia, that is, events that needed to be addressed during induction (*P* > 0.05, ***Table 5***).

**Table 5:** Comparison of complication rates

Characteristics^a^	Group P (*n*=29)	Group R (*n*=28)	*χ* ^*2*b^	*P-*value
Hypotension	8 (27.6)	3 (10.7)	2.604	0.107
Bradycardia	2 (6.9)	3 (10.7)	0.002	0.967
Hypertension	3 (10.3)	5 (17.9)	0.189	0.664
Tachycardia^c^	0 (0)	0 (0)	–	–
Injection pain	11 (37.9)	0 (0)	13.160	< 0.001^**^
Hypoxemia^c^	0 (0)	0 (0)	–	–
^a^Eligible patients were randomized into either the "propofol group" (group P) or the "remimazolam group" (group R), and categorical data were expressed as *n* (%). ^b^Chi-square (*χ*^*2*^) tests (hypotension, injection pain), and continuity correction *χ*^*2*^ test (bradycardia, hypertension) were used to compare categorical data. ^**^P < 0.001, compared with the group P.^c^Because the frequency of tachycardia and hypoxemia in both groups was 0, no statistical analysis was performed.				

## Discussion

The results of this prospective, randomized, single-blind study suggested that both remimazolam and propofol could decrease BP during general anesthesia induction in elderly patients. However, remimazolam may provide a better maintenance of hemodynamic stability, compared with propofol, potentially because of its ability to maintain SVRI levels and its minimal impact on cardiac systolic function changes.

In evaluating the depth of sedation, the BIS and the patient's state of consciousness were utilized. Although BIS was originally developed for monitoring sedation depth during propofol administration^[15]^, recent studies have demonstrated that BIS can also be employed to assess sedation depth with other sedative agents^[15–16]^, including remimazolam^[10,12,17]^. Meanwhile, Miyanishi *et al*^[18]^ noted that signs reflecting the depth of sedation (body movements, vital sign changes, *etc.*) and factors that might affect drug metabolism (regular medications, race, *etc.*) should be considered in the monitoring process. In the current study, we observed no statistically significant difference in BIS values between groups P and R at LoC or during recovery, which confirms the accuracy of BIS values in evaluating the depth of remimazolam-induced sedation.

Shirozu K *et al*^[19]^ pointed out that the magnitude of α power was almost at the same level after administration of remimazolam or propofol, but β waves were higher during sedation with remimazolam, which may result in a higher BIS value. Taking this into consideration, when BIS value dropped to 60 in the current study, remimazolam was administered at slightly higher doses than needed, indicating that patients in the remimazolam group achieved a deeper depth of anesthesia. But even so, the hemodynamic changes were still smaller in the remimazolam group. Therefore, if this factor had been taken into account, the current study would not have led to the discrepant conclusion.

An increasing number of non-cardiologists and non-sonographers are using TTE to assess cardiopulmonary function. Compared with TTE, electrical velocimetry monitoring has a limited accuracy and precision^[20]^; pulse index continuous cardiac output (PiCCO, Pulsion Medical Systems AG, Munich, Germany) is inaccurate for cardiac output (CO) measurements in hypothermic patients^[21]^; the Vigileo-FloTrac (Version 3.02, Edwards Lifesciences, Irvine, CA, USA) system and Pulsioflex (Pulsion Medical Systems AG, Munich, Germany) have unacceptable reliability, when large variations in SVR occur^[22–23]^; and the estimated continuous cardiac output (esCCO, Nihon Kohden, Tokyo, Japan) is unable to assess rapid changes in CO during surgery^[24]^. This is why we chose transthoracic ultrasonography to measure the CI, SVI, and SVRI.

Many investigators believe that TTE has advantages over other methods, as it consumes relatively little time, allows for repeated measurements, and does not require patients to be transported^[25–26]^. However, rapid hemodynamic changes occur during the induction of general anesthesia, there should not be much difference in the time when the ultrasound images were saved. Therefore, we adopted the following measures: 1) the patient's left side was padded with a 10–15 cm foam, and their left arm was extended to obtain a clearer ultrasound image; 2) the optimal location for the ultrasonic probe was determined after the first measurement; 3) the probe was placed at the marked location, and the ultrasound images were saved for further analysis; and 4) ultrasound images were saved without measurement and analysis during the induction of general anesthesia. After preliminary experiments and many times of coordinated practices, the acquisition of ultrasonic images at each time point was completed within 20 s.

Studies investigating the correlations of propofol or remimazolam with hypotension, left ventricular systolic function and SVR are limited and have yielded conflicting results^[11–12,27–28]^. Qiu *et al*^[10]^ suggested that remimazolam had a better hemodynamic stability, possibly because of its better preservation of cardiac output, while Tang *et al*^[11]^ came to the opposite conclusion. Additionally, it has been suggested that the effects may differ depending on age, with older adults experiencing a decrease in CO and younger adults experiencing a decrease in SVR^[29]^. Unlike these studies, we observed an increase in CI and SVI during anesthesia induction, potentially because of the improvement of left ventricular diastolic function, indicating that the dose and administration method of anesthetics may play crucial roles in achieving hemodynamic stability, which should be considered when selecting induction agents for elderly patients.

Although the current study has yielded some intriguing findings, several limitations should be acknowledged. First, technically, this is a single-blind study, despite our efforts to optimize the procedure. Second, it is a single-center study with a relatively small sample size. Third, the inclusion of a large number of patients who underwent transurethral laser resection of the prostate resulted in an overproportion of males in our sample. Fourth, it should be noted that BIS was more accurate in monitoring the depth of anesthesia with propofol than with remimazolam, although it was commonly used in many studies during anesthesia with remimazolam.

### Conclusions

In conclusion, the current study demonstrated that the induction of general anesthesia with either remimazolam or propofol caused a decrease in BP in elderly patients, which may be attributed to a combination of changes in left ventricular systolic function and SVR. Moreover, remimazolam maintained hemodynamic stability better than propofol during the induction, which may be attributed to its better maintenance of SVR levels, but not closely correlated with differences in cardiac systolic function.
